# Validation of an updated French national tool for reporting pharmacist interventions for all healthcare products

**DOI:** 10.1007/s11096-026-02181-5

**Published:** 2026-07-04

**Authors:** Rémi Toth, Eléonore Martin, Cordélia Salomez-Ihl, Magali Bourdelin, Julien Boyer, Virginie Chasseigne, Perrine Drancourt, Antoine Dupuis, Caroline Figeac, Aurélie Fresselinat, Stéphanie Genay, Anne-Françoise Germe, Lucie Germon, Julien Gravoulet, Audrey Janoly-Dumenil, Michel Juste, Isabelle Le Du, Morgane Masse, Céline Mongaret, Fabien Nativel, Jérôme Perrey, Chloé Rousseliere, Delphine Schmitt, Pierrick Bedouch, Bertrand Décaudin

**Affiliations:** 1 Pharmacy Department, Univ. Grenoble Alpes, CHU Grenoble Alpes, 38000 Grenoble, France; 2 CHU Lille, Institut de Pharmacie, 59000 Lille, France; 3 Université Lille, CHU Lille, ULR 7365-GRITA, Groupe de Recherche Sur Les Formes Injectables Et Les Technologies Associées, F-59000 Lille, France; 4 CNRS, UMR5525, Grenoble, INP, TIMC, CHU Grenoble Alpes, Univ. Grenoble Alpes, 38000 Grenoble, France; 5 Pharmacy Department, Centre Hospitalier de Villefranche-Sur-Saone, Villefranche-Sur-Saone, France; 6 Pharmacy Department, Centre Hospitalier de Valenciennes, Valenciennes, France; 7 Pharmacy Department, CHU de Nimes, Nimes, France; 8 Pharmacy Department, CHU de Poitiers, Poitiers, France; 9 Pharmacy Department, CHU de Bordeaux, Bordeaux, France; 10 Pharmacy Department, CHU de Clermont-Ferrand, Clermont-Ferrand, France; 11 Faculté de Pharmacie, Université de Lorraine, Nancy, France; 12 Pharmacy Department, CHU de Lyon, Lyon, France; 13 Pharmacy Department, Centre Hospitalier d’Epernay, Epernay, France; 14 Pharmacy Department, CHU de Brest, Brest, France; 15 CHU Nantes, INSERM, UMR1229, Regenerative Medicine and Skeleton, RmeS, Nantes Université, Oniris, 44000 Nantes, France; 16 Pharmacy Department, CHU de Montpellier, Montpellier, France; 17 Faculté de Pharmacie, Université de Lille, UFR3S, 3 Rue du Professeur Laguesse, BP83, F-59006 Lille Cedex, France

**Keywords:** Clinical pharmacy information systems, Equipment and supplies, Hospital, Medical devices, Pharmaceutical services, Pharmacist intervention, Pharmacy service

## Abstract

**Introduction:**

In France, two validated tools (collectively referred to as “Act-IP©”) are commonly used to report pharmacist interventions in both community and hospital pharmacy settings. These tools were designed primarily for drug-related pharmacist interventions and are less suited to those related to medical devices. To address this limitation, the French Clinical Pharmacy Society and the French Medical Device Pharmacy Society are jointly developing a rating tool that encompasses all healthcare products.

**Aim:**

To assess inter-rater agreement for "product-related problem" and "pharmacist intervention" items in a revised version of the Act-IP© during validation by internal and external panels and to evaluate the tool’s feasibility and acceptability.

**Method:**

The revised Act-IP© was based on two previously validated tools; items were revised or added to encompass interventions involving medical devices and drugs. Six expert hospital pharmacists were recruited for the internal validation, and pharmacists attending a national congress were recruited for the external validation. In both validation processes, 60 scenarios had to be rated using the new tool. Each scenario included a case report, a product-related problem, and the corresponding pharmacist interventions. The Clinical, Economic, and Organizational Scale was also used to rate the impacts of each pharmacist intervention. Inter-rater agreement was evaluated using Fleiss’s kappa (κ, interpreted according to Landis and Koch’s conventions).

**Results:**

The revised Act-IP© included 11 product-related problem items (10 revised and one new) and eight pharmacist intervention items (5 revised and one new). In the internal validation, the mean κ values were 0.63 for product-related problems and 0.77 for pharmacist interventions. In the external validation, the mean κ values were lower: 0.42 for product-related problems and 0.52 for pharmacist interventions; this reflecting time constraints, real-world assessment conditions, and greater variability among raters. The level of inter-rater agreement for the Clinical, Economic, and Organizational Scale was low and – despite the tool’s usability and applicability – highlighted variability in the raters’ judgements.

**Conclusion:**

The present findings validate the revised Act-IP© for reporting pharmacist interventions. Further work is needed to optimize the tool’s implementation and develop specific training programs for hospital pharmacists.

**Supplementary Information:**

The online version contains supplementary material available at https://doi.org/10.1007/s11096-026-02181-5.

## Impact statements

- We developed and validated a structured, multidimensional tool for standardizing documentation of clinical pharmacist interventions for medical devices.
- This revision of the Act-IP© enables the documentation, classification, and evaluation of pharmacist interventions involving drugs or medical devices.
- While the tool demonstrated strong internal reliability, the observed variability in real-world rater assessments showed that the optimization of patient safety and clinical practice will require targeted professional training and integration into electronic prescribing systems.

## Introduction

In France, hospital pharmacists are involved at each stage in the patient’s care pathway and at all levels in the healthcare product supply chain—not only drugs but also medical devices. More than two million different types of medical device (classified into over 7,000 generic groups) are currently available on the global market [1]. This diversity poses significant challenges for safe, efficient device management and makes the pharmacist’s expertise essential. A literature review published in 2017 demonstrated that pharmacists have a key role in medical device management, which can be structured into three main stages: device selection, device evaluation, and supply chain management [2]. To effectively fulfil these missions, pharmacists must interact closely with end users in all settings where medical devices are prescribed or used; these include care units and interventional, technical platforms such as operating rooms. This interactions requires the establishment of dedicated pharmacy units close by operating rooms and the presence of pharmacy staff in these areas [3, 4].

In view of this context, clinical pharmacy activities related to medical devices are growing substantially. In particular, regulatory requirements governing the documentation of medical devices are creating opportunities not only for comprehensive pharmacist-led analyses but also for the establishment of dedicated patient education initiatives [5]. These expanded responsibilities prompt pharmacist interventions, which are defined by the French Clinical Pharmacy Society (*Société Française de Pharmacie Clinique*, SFPC) as suggested changes in therapy involving one or more healthcare products [6]. With regard to drugs, two validated tools (referred to collectively as " Act-IP©”) are used routinely in France, in order to document and report pharmacist interventions in both community and hospital pharmacy settings [7, 8]. Many studies have demonstrated the scope, frequency and clinical impact of pharmacist interventions. For example, Bedouch et al*.*’s prospective study of six French hospitals analyzing 38,626 prescriptions and identified 1,800 pharmacist interventions; the high rate of acceptance (73.4%) by prescribers highlighted the interventions’ relevance and value [9]. Pharmacist interventions are also particularly valuable during critical transitions in care (such as hospital admission), as demonstrated by the results of Mongaret et al*.*’s study [10]. The performance and documentation of pharmacist interventions (i) enable accurate reporting and the traceability of the pharmacist’s contributions, (ii) enable retrospective analyses that can guide preventive or corrective actions, and (iii) make the impact of pharmacist interventions more noticeable [11]. This use is observed in many other countries, e.g. The Netherlands [12], Germany [13], and Portugal [14]. The extension of pharmacist interventions to medical-device-related practices has a number of proven, beneficial effects. Firstly, it promotes the standardized documentation of interventions. Secondly, it reinforces the value of pharmaceutical analysis. However, despite their clinical significance, the currently available rating scales were designed primarily for drug-related problems and may be less suitable for medical-device-related interventions; the specific features of medical devices’(such as traceability, administration route, frequency of use, and conditions of use) do not always align with the tools’ predefined categories.

It is noteworthy that several local initiatives have already been implemented in France. For example, Buisson et al*.* reported that Nimes University Hospital (Nimes, France) has implemented a pharmacist intervention system for peripherally inserted central catheters [15]. Similarly, Montpellier University Hospital (Montpellier, France) has implemented pharmacist interventions in the context of vacuum-assisted closure therapy [16]. The intervention tools followed the standard structure used in Act-IP©, which includes identification of the problem, the pharmacist intervention issued, and an evaluation of the potential impact on the patient. It is clear that these initiatives further emphasise the need for a standardized instrument for use with medical devices. Tracking pharmacist interventions is a major challenge in the development of clinical pharmacy activities. It constitutes a strategic lever for the implementation of activity-based or fee-for-service funding models and may ultimately contribute to the sustainable financing of clinical pharmacy. The overarching goal is to improve the patient care pathway. In France, activity-based funding mechanisms for hospital pharmacy services are evolving. The care gradation framework published in 2020 provides a notable example in which the funding of clinical pharmacy activities are integrated into the cancer care pathway [17]. In a study of patients receiving orally administered cancer drugs, Le Joncour et al*.* retrospectively evaluated the economic impact of consulting a pharmacist [18]. The researchers estimated that consultations with 579 patients generated €87,545 in hospital revenue over one year; this finding highlights the economic relevance of promoting and documenting pharmacist interventions. Lastly, integrating pharmacist intervention traceability into hospital information systems is essential for simplifying and standardizing the reporting of pharmacist interventions [19].

To address these challenges, the Act-IP© for pharmacist interventions has been adapted in a collaboration between the SFPC and the French Medical Device Pharmacy Society (*Société Pharmaceutique Française des Dispositifs Médicaux*, SPFDM). The overall goal was to extend the current Act-IP© from drugs to medical devices and thereby enable more comprehensive, consistent documentation of pharmacist interventions with regard to all types of healthcare product.

### Aim

The objectives of the present study were to (i) assess inter-rater agreement for product-related problems and pharmacist interventions with internal and external panels, (ii) validate a new version of the Act-IP© tool, and (iii) evaluate the new tool’s feasibility and acceptability.

## Method

The new tool is based on previously validated Act-IP© instruments developed by Allenet et al*.* and Vo et al*.*; the items of product-related problems and pharmacist interventions were revised to so that interventions related to medical devices could be evaluated [7, 8]. This adaptation was carried out initially by a group of four hospital pharmacists (two final-year pharmacy residents and two senior pharmacists specializing in medical devices) and included the modification of existing items and the addition of new items. The modifications were then reviewed and validated by an SFPC-SPFDM working group composed of 23 hospital pharmacists with expertise in clinical pharmacy and medical devices.

To validate the new version of the tool, the group of four hospital pharmacists created 60 clinical scenarios involving medical devices. The scenarios were based on the work of two junior physicians nearing the end of their residency; they worked from a set of specifications approved by senior pharmacists and drew inspiration from 20 clinical case scenarios developed by experts during a learned society’s a workshop at a French national conference The clinical case scenarios were based on routine clinical practice by hospital pharmacists in France (for details, see Online resource 1). The medical devices were explored with various medical specialities, care settings (critical care, care in a conventional ward, etc.), and patient profiles (elderly patients, patients with multiple comorbidities, etc.), to ensure representativity of real-world practice. Each scenario included a detailed case report, a description of the product-related problems, and the corresponding pharmacist interventions. Participants were asked to rate the problem and the intervention, using the updated rating scale. As the questionnaire had not been submitted, the participants were able to go back and make changes. No time limit was placed on the responders for individual scenarios or the assessment as a whole. A paper copy of the scoring tool was provided, and the case scenarios were displayed individually and sequentially.

The tool was validated in two phases. In the first phase (internal validation), a panel of six hospital pharmacists (three experts from the SPFDM specializing in the safe use of medical devices and three experts from SFPC specializing in the safe use of drugs, and all of whom were independent from the scenario developers) assessed the tool’s validity for all 60 scenarios. The data were entered into a Microsoft® Excel® 2019 file (Microsoft Corp., Redmond, WA, USA). To facilitate use of the tool, the panel developed a narrated slideshow as a training guide to the main changes vs. the previous tool. All the training resources are available on request to the corresponding author.

In the second phase (external validation), a panel of pharmacists, pharmacy technicians, and pharmacy residents from across France was recruited at the 2025 SPFDM congress in Bordeaux. Each member of the panel evaluated a randomly allocated block of five of the 60 scenarios by using a QR code to link to an online questionnaire (Framaforms, The Framasoft Association, https://framaforms.org/, France). All blocks of five scenarios were completed the same number of times. Furthermore, the panel members were invited to comment on the extent to which the case scenarios reflected real-life practice. A brief (5-min) training session on how to use the tool was provided to groups of two to three panel members by two of the authors (EM and RT). The target was at least six evaluations per scenario (i.e. 72 panel members), although additional recruitment (if needed) was planned through group sessions in the Auvergne–Rhône-Alpes and Hauts-de-France regions of France.

In the internal and external validation phases, each member of the panel independently assessed each scenario and used the definitions of product-related problems and pharmacist interventions provided by the tool to guide the rating process. The panel members also used the SFPC’s Clinical, Economic, and Organizational (CLEO) tool to assess the impact of the pharmacist interventions [20]. Information on the panel members’ professional practice, work setting, type of institution, proportion of activity related to medical devices and drugs, and usual practices regarding pharmacist interventions rating was also gathered. The questionnaire was designed in such as way that no data were missing.

During the internal validation phase, ease of use and acceptability were rated on a four-point Likert scale (strongly disagree; somewhat disagree; somewhat agree; strongly agree) [21]. The satisfaction questionnaire addressed the clarity of the description related to the medical devices, the items’ ease of use, overall satisfaction with the tool, willingness to use the tool in routine practice, and the training session’s perceived usefulness.

Agreement among the expert raters was assessed using Fleiss’s kappa (κ) and was calculated separately for each product-related category and each pharmacist intervention category [22]. The “drug interaction” and “selection of the administration route” items did not apply to the medical device scenarios. Furthermore, Fleiss’s κ was used to assess inter-rater agreement for use of the CLEO scale and was interpreted with regard to Landis and Koch’s conventions: κ values < 0 indicated no agreement; 0–0.20 indicated slight agreement; 0.21–0.40 indicated fair agreement; 0.41–0.60 indicated moderate agreement; and 0.61–0.8 indicated substantial agreement; and 0.81–1.00 almost perfect agreement [23].

The statistical analysis was performed using R Studio® software (version 4.4.0, R Foundation for Statistical Computing, Vienna, Austria). Quantitative variables were reported as the mean ± standard deviation when normally distributed (as verified using the Shapiro–Wilk test with *p* < 0.05) or as the median [interquartile range (IQR] if not. Qualitative variables were reported as the count (n) and percentage (%).

### Ethics approval

This study involved pharmacists who participated voluntarily by completing a questionnaire and rating scenarios. No patients or animals were involved, and no personal or sensitive data were collected. In line with French legislation, the provision of informed consent and approval by an independent ethics committee were neither required nor sought. The study was conducted in accordance with the terms of the MR-004 French national reference methodology governing the processing of anonymized personal data for research purposes.

## Results

The revised tool comprises 11 product-related problem items: 10 revised items and one new item related to “non-compliance with the healthcare product supply chain” (Online resource 2). The scale further comprises eight pharmacist intervention items: five revised items and one new item related to “optimization of the healthcare product supply chain”. The main revisions corresponded to the incorporation of medical device specifications in general and traceability requirements and features of the medical device supply chain in particular. The term “drug” was replaced with “health care product” whenever relevant.

The median percentages of routine work time dedicated to medical devices were 52.5% for the internal validation and 35% for the external validation (Table 1).

**Table 1:** Characteristics of the panel members

Characteristic	Internal validation (n = 6)	External validation (n = 84)
Profession	Pharmacists (n = 6): 100%	Pharmacists (n = 52): 62% Pharmacy residents (n = 27): 32% Pharmacy technicians (n = 5): 6%
Type of hospital	University hospital (n = 5): 83% General hospital (n = 1): 17%	University hospital (n = 45): 54% General hospital (n = 33): 39% Private clinic (n = 6): 7%
Median [IQR] number of years of professional experience (excluding residents)^a^	15.5 [11.25 –16.75]	10 [2–15]
Median percentage of routine work time dedicated to drugs	35% [0%–88.75%]	50% [4%–99%]
Median percentage of routine work time dedicated to medical devices	52.5% [1.25%–100%]	35% [0%–90%]
Panel members familiar with rating pharmacist interventions in their routine practice	4 (67%)	49 (58%)
Raters familiar with the previous Act-IP© tool	4 (67%)	39 (80%) missing data: n = 1 (4%)

^a^The pharmacy residents had between 1 and 4 years of experience overall and at least 6 months of experience in clinical pharmacy and medical devices

The ratings were concentrated into a small number of categories (Table 2). This imbalance likely influenced the κ coefficients, as agreement statistics are known to be sensitive to prevalence effects. In this context, the observed κ values for both product-related problems and pharmacist interventions should be interpreted in light of this distribution, where high agreement within dominant categories may coexist with lower overall κ values.

**Table 2:** Distributions of product-related problem items and pharmacist intervention items during the internal validation (360 ratings) and external validation (420 ratings)

	Internal validation (n = 360 ratings), n (%)	External validation (n = 420 ratings), n (%)
*Product-related problem item*		
Non-compliance with guidelines, or a contraindication	81 (22.5%)	87 (20.7%)
Untreated indication	28 (7.8%)	28 (6.7%)
Subtherapeutic dosing/insufficiently frequent replacement	12 (3.3%)	16 (3.8%)
Supratherapeutic dosing/overly frequent replacement	28 (7.8%)	31 (7.4%)
Healthcare product not indicated	27 (7.5%)	33 (7.9%)
Drug interaction*	10 (2.8%)	5 (1.2%)
Adverse drug reaction	29 (8.1%)	32 (7.6%)
Inappropriate administration route and/or use	47 (13.1%)	81 (19.3%)
Failure to receive the treatment	43 (11.9%)	34 (8.1%)
Required monitoring not performed	13 (3.6%)	20 (4.8%)
Non-compliance with the healthcare product supply chain	42 (11.7%)	53 (12.6%)
*Pharmacist interventions*		
Addition (initiation of a new treatment)	33 (9.2%)	44 (10.5%)
Treatment discontinuation	17 (4.7%)	21 (5.0%)
Substitution/switching	136 (37.8%)	139 (33.1%)
Selection of the administration route *	3 (0.8%)	15 (3.6%)
Therapeutic monitoring	18 (5.0%)	24 (5.7%)
Optimization of administration/use	63 (17.5%)	103 (24.5%)
Dosing adjustment	41 (11,4%)	22 (5,2%)
Optimization of the healthcare product supply chain	49 (13.6%)	52 (12.4%)

^*^This item was not related to medical devices

Fleiss’s κ indicated substantial overall agreement for product-related problems (κ = 0.630) during the internal validation, with values ranging from 0.271 to 0.962 (Table 3). Substantial agreement was also measured for pharmacist interventions (κ = 0.770), with values ranging from 0.581 to 0.938 (Table 4). In the external validation, the κ coefficients were lower. Agreement for product-related problems was moderate (κ = 0.419), with values ranging from 0.266 to 0.712. Similarly, the agreement for pharmacist interventions was moderate (κ = 0.523), with values ranging from 0.344 to 0.720.

**Table 3:** Inter-rater agreement for coding product-related problems using Fleiss’s κ (n = 60 scenarios) for the internal and external validations (κ values: 0–0.20 = slight agreement, 0.21–0.40 = fair agreement, 0.41–0.60 = moderate agreement, 0.61–0.80 = substantial agreement, 0.81–1.00 = almost perfect agreement)

	Inter-rater agreement	
Product-related problem	Internal validation (6 ratings per scenario, 360 ratings in total)	External validation (7 ratings per scenario, 420 ratings in total)
Non-compliance with guidelines, or a contraindication	0.506	0.241
Untreated indication	0.768	0.579
Subtherapeutic dosing/insufficiently frequent replacement	0.448	0.350
Supratherapeutic dosing/overly frequent replacement	0.783	0.675
Healthcare product not indicated	0.271	0.266
Drug interaction	/*	/*
Adverse drug reaction	0.962	0.650
Inappropriate administration route and/or use	0.418	0.210
Failure to receive the treatment	0.646	0.307
Required monitoring not performed	0.920	0.685
Non-compliance with the healthcare product supply chain	0.903	0.712
Overall agreement	0.630	0.419

^*^Item not related to medical devices

**Table 4:** Inter-rater agreement for coding pharmacist interventions using Fleiss’s κ (n = 60 scenarios) for internal and external validation (κ values: 0–0.20 slight agreement, 0.21–0.40 fair agreement, 0.41–0.60 moderate agreement, 0.61–0.80 substantial agreement, 0.81–1.00 almost perfect agreement)

	Inter-rater agreement	
Pharmacist interventions	Internal validation (6 ratings per scenario, 360 ratings in total)	External validation (7 ratings per scenario, 420 ratings in total)
Addition (initiation of a new treatment)	0.833	0.636
Treatment discontinuation	0.938	0.566
Substitution/switching	0.820	0.548
Selection of the administration route	/*	/*
Therapeutic monitoring	0.883	0.720
Optimization of administration/use	0.581	0.374
Dosing adjustment	0.697	0.344
Optimization of the healthcare product supply chain	0.863	0.707
Overall agreement	0.770	0.523

^*^Item not related to medical devices

All scenarios were successfully assessed with regard to the clinical, economic and organizational impact of the pharmacist intervention, using the CLEO scale. For both the internal and external validation phases, the values of Fleiss’s κ were corresponded to poor to fair agreement, according to Landis and Koch classification (Table 5).

**Table 5:** Overall inter-rater agreement for the three dimensions of the CLEO scale. (Fleiss’s κ: 0–0.20 = slight agreement, 0.21–0.40 = fair agreement, 0.41–0.60 = moderate agreement, 0.61–0.80 = substantial agreement, 0.81–1.00 = almost perfect agreement)

CLEO scale	Internal validation (360 ratings)	External validation (420 ratings)
Clinical impact	0.166	0.134
Economic impact	0.244	0.359
Organizational impact	0.0834	0.0225

**Fig. 1:**
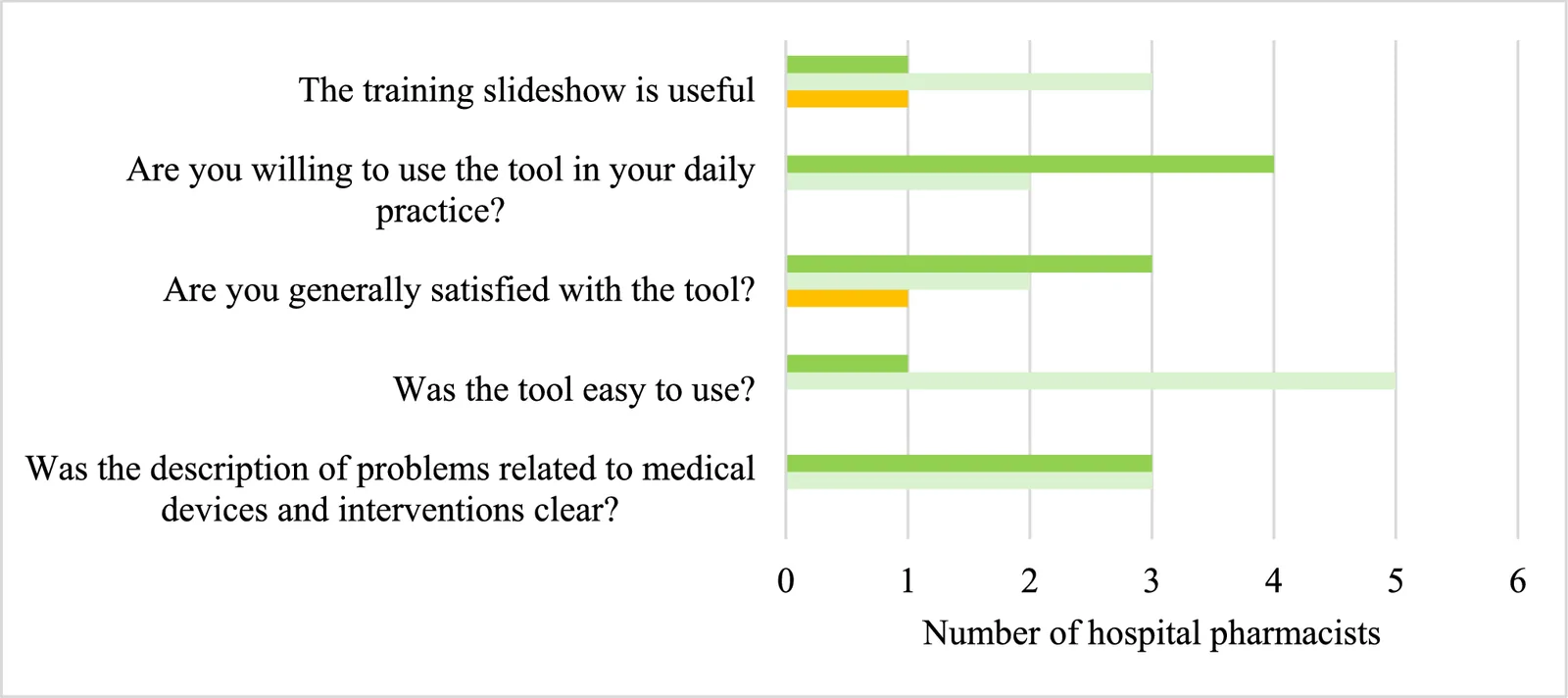
User satisfaction survey of the six hospital pharmacists involved in the internal validation. Strongly agree = dark green; somewhat agree = light green; somewhat disagree = orange

Regarding the satisfaction questionnaire completed during the internal validation phase, three of the six experts somewhat agreed that the problem description was clear, while the remaining three strongly agreed (Fig. 1). One expert rated the tool as “very easy to use”, whereas the remaining five rated it as “easy to use”. With regard to willingness to incorporate the tool into their daily clinical practice, two experts expressed moderate agreement, while four indicated strong agreement. The training support was generally perceived to be useful: one respondent strongly agreed, three somewhat agreed, and one somewhat disagreed.

## Discussion

All scenarios were successfully assessed using the modified Act-IP©; hence, the tool’s application to medical devices was feasible. The present results appear to be consistent, with (i) substantial agreement in the internal validation among experienced operators and (ii) moderate agreement in the external validation among a broader group of users less familiar user with the tool. We were able to valid the integration of all healthcare products into the new Act-IP® but the difference between the user groups highlights the need for training for pharmacists who are less familiar with the tool (such as those specializing in medical devices). However, this need was not unexpected, and we therefore developed a bespoke training resource (available as a commented slideshow) for consultation prior to scenario rating during the internal validation phase. Most of the experts involved in the internal validation reported that this resource was useful. Furthermore, with regard to the external validation, two of the authors provided congress participants with a brief training session.

One of the modified Act-IP©’s main strengths is its foundation on well-established instruments used routinely in France; it is immediately familiar to clinical pharmacists accustomed to use of the existing, drug-only version. Use of the Act-IP© as the basis for this work was appropriate and justifiable because it had already been applied to the assessment of pharmacist interventions related to medical devices [8]. However, the adaptation of certain items was required for other situations, which prompted the present work and the work by Hami et al*.* [24]. To ensure methodological consistency with previous validations of the Act-IP©, Fleiss’s κ was calculated. This statistic is appropriate for assessing agreement for nominal data across multiple raters, assuming that categories are mutually exclusive and not intrinsically ordered. However, the interpretation of Fleiss’s κ is sensitive to prevalence and potential bias.

Another major strength of the modified Act-IP© is that it can be used to evaluate issues related to drugs or medical devices and thus avoids the need for two separate tools. The tool was validated by using much the same methodology as used by Allenet et al*.* and Vo et al*.*, i.e. the assessment of inter-rater agreement across 60 cases scenarios with a similar distribution of product-related items and pharmacist intervention items [7, 8]. Our κ values for device-related problems (0.63 in the internal validation and 0.419 in the external validation) were lower than the value found in Allenet et al*.*’s hospital-based study (0.76). Nevertheless, our values indicate that the level of agreement was moderate to substantial. Our κ values for pharmacist intervention items (0.77 in the internal validation, i.e. substantial agreement, and 0.523 in the external validation, i.e. moderate agreement) were also lower than the value of 0.89 (i.e. almost perfect agreement) reported by Allenet et al*.* The lower levels of agreement observed in our study might be due (at least in part) to our inclusion of a larger, more heterogeneous sample that consisted of 84 pharmacy professionals (pharmacy residents, clinical pharmacists, and other pharmacists) from diverse settings. Furthermore, the κ values obtained during the external validation may have been influenced by contextual constraints, such as limited time available for responses, potentially noisy environments, and the inability to modify responses after submission. Although these factors are known to reduce the value of Fleiss’s κ, they also highlight the relevance of the present work; in contrast to the controlled settings used in earlier validation studies, our external validation phase more closely reflected real-world practice.

The lower level of agreement might also be due in part to the inappropriate inclusion of items that should not have been selected for rating medical-device–related scenarios. Specifically, the “selection of the administration route” and “drug interaction” items were intended for pharmacist interventions related to drugs and so were not applicable to the 60 device-related scenarios included in the present study. However, these items were still selected during the rating process. The “drug interaction” item was selected in 10 (2.8%) of the 360 ratings during the internal validation and in 5 (1.2%) of the 420 ratings during the external validation. Similarly, the “selection of the administration route” item was selected in 3 (0.8%) of the 360 ratings during the internal validation and in 15 (3.6%) of the 420 ratings during the external validation.

The distribution of the ratings over the tool’s items might also have influenced the κ values. For product-related problems, a large proportion of the ratings was concentrated on a small number of items. For example, “non-compliance with guidelines or contraindications” accounted for 81 (22.5%) of the 360 votes during the internal validation, and 87 (20.7%) of the 420 votes during the external validation. This concentration suggests that some items were overused and others were underused, which might also have contributed to lower levels of inter-rater agreement. For example, "substitution/switching” accounted for 136 (37.8%) ratings during the internal validation and 139 (33.1%) during the external validation. In contrast, “subtherapeutic dosing/insufficiently frequent replacement” was chosen only 12 (3.3%) times during the internal validation and 16 (3.8%) times during the external validation, and “treatment discontinuation” accounted for only 17 ratings (4.7%) during the internal validation and 21 votes (5.0%) during the external validation. A similar pattern was observed for pharmacist interventions,

The inter-rater agreement for CLEO ratings was low, with κ values indicating slight or fair agreement. There are several possible explanations for this low level of agreement. Pharmacists who usually work with medical devices have little or no knowledge of the CLEO framework. Some of the study participants were using the framework for the first time and so needed time to familiarize themselves with it. Conversely, pharmacists who were familiar with the tool had a more limited understanding of the potential clinical, economic and organizational impact of the medical devices in the scenarios. The absence of information on the price of the medical devices might also have influenced the value of κ for the economic impact of the pharmacist intervention.

A major strength of our study lay in the joint involvement of the SFPC and the SPFDM in the Act-IP© modification process; the tool therefore reflects the collective expertise of the pharmacist community in France and thus gained in scientific and professional legitimacy. Another key strength was the inclusion of 84 pharmacy professionals from diverse backgrounds (including residents, pharmacists and pharmacy technicians) and from the private and public sectors in the external validation phase. This diversity boosts the relevance, generalizability and robustness of the modified Act-IP©.

The Act-IP© has now been formally approved for nationwide implementation in France. The inclusion of supply–chain–related categories – namely “non-compliance with the healthcare product supply chain” for product-related problems and “optimization of the healthcare product supply chain” for pharmacist interventions – reflects increasing concerns regarding traceability and the overall management of healthcare product logistics. The Act-IP©’s potential transfer to other European settings should take into account national differences in regulations, the legal status of healthcare products (e.g., differences in the pharmacists’ regulatory responsibilities and duties regarding sterile medical devices), and heterogeneity in supply chain organisation and traceability requirements for implantable devices. Despite these regulatory and organisational differences, Act-IP© was designed to support and promote clinical pharmacy activities in a variety of practice environments. A recent systematic review of the literature highlighted significant weaknesses in the supply chain of implantable medical devices – especially in traceability and patient information [25]. Pharmacists have a pivotal role in strengthening traceability systems in general and those dealing with implantable medical devices and blood products in particular [26–28]. According to the European Union’s Regulation 2017/745, a medical device manufacturer is legally obliged to maintain accurate traceability and to provide patients with relevant information. The failure of a traceability system is likely to compromise device vigilance or pharmacovigilance systems and thus hinder the detection and management of adverse events, with important clinical consequences. Similarly, inappropriate initiation of the ordering process might disrupt continuity of supply, which would also have important clinical implications. In this context, pharmacist interventions are essential for preventing disruption and ensuring the safe, efficient management of healthcare products. Pharmacists working in clinical departments also contribute to the appropriate use of medical devices by consulting directly with patients [5, 29]. This positive feedback is encouraging and reflects the literature on the development of tools for rating pharmacist interventions related to medical-device-related processes. According to the literature, structured pharmacist involvement in these processes is associated with cost savings in surgical settings, fewer device-use errors, and better care for patients with peripherally inserted central catheters [30, 31]. Between 2018 and 2021, Tournayre et al*.* conducted a retrospective, observational study of pharmacist interventions related to requests for peripherally inserted central catheters: 59.9% of the 5,503 requests required at least one pharmacist intervention [32]. The acceptance rate for these pharmacist interventions was high. Taken as a whole, these findings highlight the high frequency and clinical relevance of medical-device-related pharmacist interventions and emphasize the need for a structured, standardized method of documenting, classifying and assessing these interventions (e.g. by adapting the current Act-IP© tool for application to pharmacist interventions related to all type of medical devices).

One limitation of the present study relates to the fact that the scenarios developed to validate the tool addressed medical-device-related problems but not drug-related problems. This might question the tools’ applicability to drug-relating problems. However, this limitation is unlikely to compromise the validity of the tool because the original version of the tool was specifically developed for drug-related problems. One can reasonably assume that these changes had a minor impact on ratings of pharmacist interventions related to drugs, which had previously been validated in two other studies (as illustrated by the example given in the Online resource 3). The scenarios were designed to reflect the range of situations encountered in a pharmacist’s clinical practice. Differences between scenarios were not expected to influence agreement between evaluators faced with the same scenario, except when the situation had already been encountered by the evaluator. This approach is in line with the method used in previous validation studies. The only substantial modification to the conceptual framework that might have influenced how pharmacists rate drug-related pharmacist interventions concerned the healthcare product supply chain. We plan to assess the performance of the revised Act-IP© in mixed scenarios, i.e. with pharmacist interventions related to medicines and to medical devices. Work on this study began after a recent workshop at the national SFPC conference evaluated the ability of pharmacists to assess mixed medicine-medical device scenarios. Despite these limitations, the two-step validation process demonstrated the Act-IP©’s robustness and applicability. For broader implementation, further evaluation within electronic prescribing systems and targeted training for pharmacists and pharmacy residents will be required. Several methodological factors might have influenced our findings, including potential selection bias related to congress-based recruitment, a brief training session conducted in a noisy environment, and the absence of test–retest reliability or responsiveness measures. To address these limitations, further validation of a mixture of medical device and drug scenarios by experienced, versatile hospital pharmacists would be valuable. Lastly, we did not formally assess the revised Act-IP©’s responsiveness or sensitivity to change because the study’s primary objective was to evaluate the implementation of the tool. Prospective evaluation of these aspects during Act-IP©’s deployment within specific organisations would be valuable. Future research could also usefully investigate the extent to which the tool is able to detect differences in pharmacist intervention profiles between institutions or changes over time.

## Conclusion

The revised Act-IP© (comprising 11 items on product-related problems and 8 items on pharmacist interventions), demonstrated substantial internal reliability and moderate external validity (κ = 0.42 for product-related problems and 0.52 for pharmacist interventions); this probably reflected rater variability and real-world constraints on the assessments. All the scenarios were successfully evaluated, which confirmed the revised Act-IP©’s feasibility and applicability in clinical practice and confirmed the value of extension to all healthcare products. This modification of the widely used, nationally recognized Act-IP© ensures continuity and easier adoption by clinical pharmacists. Our study also highlighted the clinical relevance of medical device–related interventions and the need for a structured approach, while the lower level of inter-rater agreement observed with the CLEO scale underscored the importance of targeted training—particularly for less experienced users. Future work will focus on three areas that are essential for facilitating the tool’s implementation and consistent, standardized use in routine daily practice: (i) large-scale dissemination, (ii) development of structured training programmes for pharmacists, and (iii) integration of the tool into pharmacy information systems. The ultimate goal is to establish Act-IP© as the reference standard for professional practice in both public- and private-sector clinical pharmacy settings.

## Supplementary Information

Below is the link to the electronic supplementary material.

Supplementary file1 (DOCX 52 KB)

Supplementary file2 (DOCX 36 KB)

Supplementary file3 (DOCX 20 KB)

## Data Availability

All data generated or analyzed during this study are available for download as supporting information.
