# Supplementary material for: Validation of an updated French national tool for reporting pharmacist interventions for all healthcare products
Source: Int J Clin Pharm. 2026 Jul 4;48(5):2015–25. doi: 10.1007/s11096-026-02181-5 (PMC13569509; doi:10.1007/s11096-026-02181-5)
Supplement: Supplementary file 1 [file 11096_2026_2181_MOESM1_ESM.docx]

**Online Resource 1: Scenarios used for validation of the revised ActIP^®^**

***This table contains the complete set of case scenarios sent to pharmacists for rating during the validation study. Each scenario includes the clinical context, the product-related problem identified by the pharmacist, and the corresponding pharmacist intervention.***

| **No.** | **Clinical context** | **Product-related problem identified by the pharmacist** | **The pharmacist intervention** |
| --- | --- | --- | --- |
| **1** | A nurse calls about Mr. Y, who has been receiving AVANCE SOLO mini-negative pressure wound therapy (NPWT) for three days. The device has been sounding an "air-leak" alarm for one hour. The NPWT has been temporarily discontinued and the nurse requests a replacement dressing. | The pharmacist visits the patient, together with the nurse. The dressing is still in place and the device is indeed indicating a leak, despite several attempts to restart it. During the discussion with the nurse, the pharmacist determines that the nurse does not know how to apply the NPWT device correctly. | The pharmacist suggests finding and sealing the leak before changing the dressing. The pharmacist listens with a stethoscope around the dressing area. Once the leak has been identified, the nurse seals it with a barrier film, according to the pharmacist's recommendations. |
| **2** | A taurolidine-citrate lock is prescribed by the nutritionist as primary prevention for Mrs. G., a patient with a Broviac-type tunneled central venous catheter for long-term parenteral nutrition. The following information is found in the patient's file: hospital admission for sepsis, suspected Broviac catheter entry point, blood cultures ongoing, empirical IV antibiotic therapy initiated. | The taurolidine-citrate is a Class III medical device and is not appropriate for the curative treatment of catheter-associated infections. The pharmacist identifies the use of this device as non-compliance with the institution's guidelines. | The pharmacist suggests replacing the taurolidine-citrate lock with an antibiotic lock that is appropriate for the identified pathogen, in addition to systemic antibiotic therapy. |
| **3** | Mr. T, 61, is scheduled for surgery in three days' time. He has been prescribed a dual-chamber defibrillator for an atrioventricular conduction disorder. | A shortage at the manufacturer means that the defibrillator is not in stock: it is impossible to fill the prescription. | The pharmacist contacts the surgeons and suggests prescribing a defibrillator produced by another manufacturer. |
| **4** | Mr. P has urinary tract problems for which a long-term indwelling urinary catheter has been prescribed. | The prescription is incomplete because the frequency of catheter replacement is not specified. | Given the risk of inappropriate use, the pharmacist suggests that the physician amend the prescription to specify "change every 28 days." |
| **5** | Mr. P has had an indwelling catheter for six weeks, due to benign prostatic hyperplasia. The urinary catheter was changed on August 26. Mr. P consults on August 28 for febrile hematuria following a fall. A urine culture performed on August 28 reveals *E. faecium* at 10^5^ CFU/mL. A course of antibiotics (according to the antibiogram results) has been initiated. | The antibiotic treatment should have been accompanied by a change of the urinary catheter after 24 hours of effective antibiotic therapy; this was not done for Mr. P. | The pharmacist recommends that the physician immediately changes Mr. P's catheter and reviews the replacement frequency. |
| **6** | Mr. D has been admitted to a nephrology ward. A peripheral catheter was prescribed six days previously because Mr. D was unable to swallow his medication. However, for the past four days, Mr. D has been able to take his medication again, and the vascular access is no longer being used. | The patient still has vascular access, even though it has not been used for four days. Access routes must be reassessed regularly, and an unused catheter must be removed if it is no longer indicated. | The pharmacist suggests discontinuation (removal) of the unused catheter. |
| **7** | Mr. C, 85, has been admitted to the geriatric ward for shortness of breath and deterioration in his general condition. Two years previously, the onset of ventricular arrhythmia prompted the implantation of a pacemaker. The attending physician prescribed regular follow-up with a cardiologist. | During the post-admission medication reconciliation, the pharmacist notes that Mr. C never attended his cardiac implant check-ups because "he didn't seen the point". | The pharmacist informs the patient's geriatrician and suggests to Mr. C that he should see a cardiologist and have the pacemaker checked. |
| **8** | Mrs. Z, 84, has been admitted for a total knee replacement. The pharmacy receives a traceability form after the operation. | The implanted medical device's traceability information has been recorded in the wrong patient's electronic records. | The pharmacist asks the department to correct the traceability record and assign it to the appropriate patient. |
| **9** | Ms. Y is simultaneously prescribed an irrigation dressing (Hydroclean Advance) and a superabsorbent dressing (Resposorb) for a venous ulcer that is fully fibrinous and highly exudative. The nurse plans to apply both an irrigation dressing (to clean the fibrin) and a super-absorbent dressing (to manage the exudate). | Applying a superabsorbent dressing over an irrigation dressing prevents the latter from functioning properly by absorbing the fluid that is supposed to clean the wound. | The pharmacist suggests discontinuing the irrigating absorbent dressing and prioritizing management of the exudate. |
| **10** | Ms. W has been admitted to the intensive care unit for severe bradycardia and is receiving an infusion of isoprenaline. The intensive care intern asks the pharmacist for advice regarding the infusion equipment. | The pharmacist recognizes that isoprenaline is highly photosensitive and thus will degrade rapidly if a transparent administration set is used. | The pharmacist recommends using an opaque administration set (brown or black syringe and tubing) or a light-blocking sleeve over a transparent administration set. |
| **11** | Ms. U has been admitted to the digestive surgery department. During her stay, a peripherally inserted central catheter (PICC) in the left arm is prescribed, in order to administer parenteral nutrition and IV antibiotic therapy for 7 days. The patient's medical history includes left breast cancer and axillary lymph node dissection in early 2024. | Previous axillary lymph node dissection can lead to lymphedema, which contraindicates the placement of a PICC in the ipsilateral arm. | The pharmacist calls the department to report the contraindication and asks for the prescription to be changed to indicate that the PICC must be placed in the right arm. |
| **12** | Mrs. T., 82, has been admitted to the geriatric medicine department for severe malnutrition. Enteral nutrition via a nasogastric tube is prescribed, with a planned duration of at least 10 days. The physician prescribes a PVC catheter, which is readily available in the department. | During the validation step, the pharmacist notes that PVC is poorly biocompatible and thus is indicated for very short-term use (24-48 hours) only. PVC is not a suitable material for a catheter that is expected to remain in place for at least 10 days. | The pharmacist contacts the medical team and recommends replacing the PVC catheter with a polyurethane catheter, which should be better tolerated and is suitable for long-term use. |
| **13** | Ms. T accidentally received a morphine bolus during patient-controlled anesthesia. The nursing team calls the pharmacy for assistance in adjusting the infusion setup. | Various infusion devices and techniques are used in the department. The infusion set most frequently used includes safety features to prevent the accidental administration of boluses. | The pharmacist adjusts the infusion system and suggests using a dedicated infusion line with an anti-siphon valve and a non-return valve. |
| **14** | Ms. S has been admitted to hospital for a pelvic collection following laparoscopic abdominoperineal resection for rectal cancer and colostomy. Discharge to home is planned after three weeks in hospital. The following prescription is written:  Coloplast Laboratory Convex fit flex support, diameter 50, ref.169830, 3 boxes  Pouches ref.122430 1 box  Paste ref. 26501 1 tube  Non-woven, non-sterile compresses 10x10 1 pack. | The duration of treatment and frequency of use/replacement are not indicated for any of the medical devices on the prescription; this might result in a shortage of material or, conversely, an excess amount of material left over. | The pharmacist asks the physician to modify the prescription to specify quantities, the duration of treatment, and references. |
| **15** | Mrs. S, 83, has a pacemaker fitted and undergoes atrioventricular junction ablation in the operating room. The pharmacy receives a traceability form after the operation. | The wrong batch number for the "MICRA VR" pacemaker is recorded in the patient's electronic records. | The pharmacist asks the department to correct the batch number. |
| **16** | Ms. R., 38, has a history of breast cancer and undergoes breast reconstruction with silicone implants after a mastectomy.  The operation and the immediate postoperative course are uneventful. | Two months after the operation, Ms. R presents at the oncology day hospital with increasing pain, redness, and local tension around the implant. The findings are consistent with a chronic inflammatory reaction (no infection) probably related to poor biocompatibility of the silicone material. | The pharmacist suggests analyzing the type of silicone used and seeking an allergist’s opinion. |
| **17** | Ms. R, a 58-year-old patient with type 1 diabetes using an external insulin pump, has been hospitalized in a general ward for a urinary tract infection and then transferred to surgery for an appendectomy.  She uses her pump to continuously adjust her insulin dose and is monitored regularly by a diabetologist. | During the transfer to surgery, the pharmacist notices that the prescription for the pump's insulin has not been renewed. | The pharmacist alerts the surgical team and has the pump prescription renewed. |
| **18** | Mrs. J, 89, has been admitted from a nursing home to the geriatric ward for a short stay, due to uncontrolled diarrhea and a sacral pressure ulcer that was not healing, with deterioration of the surrounding area. The nurses contact the pharmacy because there is not enough Flexi-seal in stock to last the weekend (Flexi-Seal is a balloon-type fecal management system designed to channel liquid and semi-liquid stools into a collection bag). An urgent prescription for daily Flexi-seal replacement is issued. | The frequency of replacement is too high. The tube is being changed daily, whereas it can remain in place for up to 28 consecutive days. | The pharmacist recommends reducing the replacement frequency to once every 2 to 3 weeks. |
| **19** | Ms. H has type 2 diabetes and is being treated with insulin for two weeks because of poor glycemia control during an infection (erysipelas), for which she is currently receiving antibiotics. The infectious disease specialist prescribed the Freestyle Libre 2 subcutaneous glucose sensor. The level of glycated hemoglobin (HbA1C) measured during the week is 7%. Ms. No consultations with an endocrinologist are mentioned in the medical records. Ms. H is scheduled for discharge in one week and will continue to use the subcutaneous glucose sensor. | The Freestyle Libre 2 device is reimbursed for patients aged 4 years or over with type 2 diabetes and poor glycemia control (HbA1C > 8%) and who are treated in the community with non-intensive insulin therapy (<3 injections per day).  The device is not indicated for patients undergoing short-term insulin therapy. | The pharmacist contacts the prescriber to suggest an alternative method of glycemia monitoring. |
| **20** | Mrs. G, 90, has an exudative leg wound for which Coloplast Comfeel Plus Transparent REF 335330 dressings were prescribed, with a change every 10 days. | The manufacturer's instructions state that the dressing should be changed after no more than 7 days. The dressing is being left in place too long. | The pharmacist suggests increasing the frequency of dressing changes. |
| **21** | Mrs. G, 76, has suffered from Parkinson's disease for many years. The neurologist initiates treatment with Scyova® (foslevodopa/foscarbidopa). The nurse asks the pharmacist about the pump prescribed by the physician. | During a medication reconciliation, the pharmacist notices that the Vyafuser pump required for the correct administration of Scyova® has not been prescribed: a different pump has been prescribed. | The pharmacist suggests replacing the prescribed pump with the Vyafuser pump. |
| **22** | Ms. C, 45, has been admitted for an urinary tract infection requiring a two-week course of IV antibiotic therapy. | During the clinical staff meeting, the pharmacist learns that the catheter has been left in place for eight days. A replacement should have been prescribed after seven days. | The pharmacist requests replacement of the catheter. |
| **23** | Ms. C, 45, has been admitted for a bone and joint infection requiring a 12-week course of IV antibiotics. | The medication is administered via a peripheral catheter, which is replaced every 96 hours. For a long (12-week) course of treatment, placement of a peripherally inserted central catheter is recommended for reducing venous damage. | The pharmacist suggests placement of a peripherally inserted central catheter. |
| **24** | Mrs. B, 81, has been admitted to a geriatric ward for continuous cardiac monitoring after experiencing atrial fibrillation. Adhesive ECG electrodes are used. | On day 4, the patient develops diffuse erythema with oozing beneath several electrodes. The suspected diagnosis is irritant or allergic contact dermatitis, probably caused by the adhesive or conductive gel. | The pharmacist suggests changing to hypoallergenic adhesive electrodes without rosin. |
| **25** | Mrs. B, 81, has been admitted for continuous cardiac monitoring after experiencing atrial fibrillation. She has developed persistent contact dermatitis under the (hypoallergenic) ECG electrodes. | The pharmacist initially suggested using hypoallergenic electrodes, which reduced the intensity of the contact dermatitis. However, the contact dermatitis persisted to a moderate degree. | The pharmacist recommends limiting prolonged exposure by changing the electrode sites regularly. |
| **26** | Ms. B, 21, has malabsorption syndrome and is receiving enteral nutrition through a nasogastric tube. The physician prescribes tube replacement every 10 days for an estimated duration of at least 6 months. | Long-term nasogastric tube use is not appropriate in Ms. B's situation. | The pharmacist recommends switching to a device suited to long-term use, such as a gastrostomy button. |
| **27** | Ms. B, 21, has anorexia and is receiving enteral nutrition through a nasogastric tube. The physician prescribes tube replacement every 2 days for approximately 15 days. | Frequent replacement of a nasogastric tube increases the likelihood of complications and therefore is not recommended. | The pharmacist recommends reducing the frequency of tube replacement. |
| **28** | Mrs. A., 67, suffers from severe osteoarthritis of the knee and is scheduled for total knee replacement in three days' time. | The pharmacist receives notification that the knee prosthesis is not available because its CE marking under Regulation (EU) 2017/745 (MDR) has been suspended. No stock is available and a resupply date has not been provided. Surgery must therefore be postponed. | The pharmacist alerts the surgical team and suggests an alternative prosthesis. |
| **29** | Ms. A., 27, has a wound with heavy exudate. It is being treated with a dry compress secured with Tensoplast® adhesive tape. | The dry compress does not allow adequate exudate management and increases the risk of maceration and delayed healing. | The pharmacist suggests using an exudate-absorbing dressing, such as hydrofiber or alginate. |
| **30** | Mrs. X has chronic kidney disease and is on dialysis via an arteriovenous fistula. A peripherally inserted central catheter (PICC) is prescribed for the administration of a one-week course of IV antibiotics. | PICC insertion - even in the contralateral arm - is contraindicated by the presence of an arteriovenous fistula, due to the risk of thrombosis. | Based on the treatment duration and need to preserve the patient's veins, the pharmacist suggests an alternative (peripheral) type of venous access. |
| **31** | Mr. Z is being followed up for a neurological condition and is monitored with MRI on a regular basis. He is prescribed with a custom cranial reconstruction implant with titanium reinforcement. | The choice of titanium is inappropriate; it might create MRI artifacts that could interfere with diagnosis and delay treatment. | The pharmacist suggests using an MRI-compatible custom cranial implant. |
| **32** | Mr. Z has been admitted to the intensive care unit. A triple-lumen central venous catheter has been placed for the short-term IV administration of various substances (norepinephrine, midazolam, sufentanil, antibiotics, potassium chloride, insulin, and parenteral nutrition). Catheter replacement is not possible, due to coagulation disorders and a lack of alternative access. | Due to the lack of low-dead-space access, the infusion setup increases the likelihood of flow disturbance and physicochemical incompatibilities. | The pharmacist recommends modifying the setup by replacing bidirectional valves with a multi-lumen extension equipped with a non-return valve and a low dead space. |
| **33** | Mr. Z has been admitted to the intensive care unit. A triple-lumen central venous catheter has been placed for the short-term IV administration of a number of substances: norepinephrine, midazolam, sufentanil, antibiotics, concentrated potassium chloride, insulin, parenteral nutrition, etc.  It is not possible to change the catheter because it is essential, there is no other suitable insertion site, and the patient has a coagulation disorder. The nurses in the intensive care unit ask the pharmacy to modify the current infusion setup: a catheter base equipped with a bidirectional valve and a distal line equipped with a manifold. | The is a major risk of flow disruption with drugs with a narrow therapeutic window and physicochemical incompatibility.  The infusion set on the catheter does not have sufficient access close to the base or a sufficiently low dead space for management of the infusion risk. | The pharmacy team collaborates with the nursing team to define appropriate infusion lines and connection sites. |
| **34** | Mr. Y presents with contact dermatitis after the use of an adhesive dressing. A hypoallergenic dressing is prescribed. | Discussions with the prescriber confirm that Mr. Y is suffering from non-allergic irritant dermatitis. | The pharmacist suggests optimizing the application conditions by ensuring that the skin is clean and dry before placement of the dressing. |
| **35** | Mr. X, 72, has undergone a partial laryngectomy and then tracheostomy. He reports discomfort and irritation of the mucosae of the upper airways. | The pharmacist notes that a humidification device has not been prescribed. | The pharmacist suggests prescribing an air humidifier or a humidification chamber for connection to the tracheostomy tube. |
| **36** | Mr. W is scheduled to undergo total hip replacement, following a femoral shaft fracture and cup loosening.  The ward sends the prescription to the pharmacy, requesting delivery of a set of different sizes by the supplier. This includes the stem, the insert, and a replacement acetabulum with screws | A femoral head is not mentioned in the prescription, even though this is a total hip replacement. There is no permanent stock at the hospital for the items on the prescription. | The pharmacist checks with the prescriber and adds a set of femoral heads to the prescription. |
| **37** | On the advice of a pulmonologist, Mr. T (a 60-year-old patient with asthma) has been admitted to a nursing home for long-term treatment with bronchodilators and inhaled corticosteroids via a nebulizer. Despite the initiation of this treatment, the patient's respiratory symptoms persist. | The pharmacist notes that the patient's respiratory symptoms have not been controlled and observes that the patient's use of the nebulizer is incorrect. | The pharmacist shows the patient how to use the nebulizer properly. |
| **38** | Mr. R has been admitted to the intensive care unit. He has a kidney transplant and is taking an immunosuppressant. He is unable to swallow, and so the immunosuppressant is being administered intravenously via a PVC tube. | PVC should not be used with the immunosuppressant, which is adsorbed onto the tubing. This incompatibility can result in incorrect administration. | The pharmacist identifies the error and suggests replacing the PVC tube with a polyethylene extension (inert with regard to the immunosuppressant) to ensure adequate bioavailability. |
| **39** | Mr. P suffers from bilateral sensorineural hearing loss requiring the insertion of a cochlear implant. During the staff meeting on preparation of the operating rooms, the pharmacy technician learns that a problem has delayed the operation. A temporary operating theatre nurse assumed that the implant was in stock in the operating room, and so the device was not ordered. | The pharmacist notes that the ordering procedure had not been followed correctly and that the implantable medical device was not available because a prescription had not been sent to the pharmacy. | The pharmacist asks the surgeon to issue a prescription to for the cochlear implant. |
| **40** | Mr. P has a transplant and is receiving the IV-administered immunosuppressant IV cyclosporine (Sandimmun®). | The drug is administered via standard PVC tubing, and the compatibility between the two has not been checked. Cyclosporine formulated with polyoxyethylated triglycerides (Cremophor EL) can adsorb onto PVC and extract plasticizers from this plastic. The use of PVC tubing should therefore be avoided. | The pharmacist detects the error during the validation stage and recommends the use of a polypropylene syringe with a polyethylene extension (i.e. inert, plasticizer-free materials). |
| **41** | Mr. L. has been hospitalized for an intestinal obstruction requiring nasogastric intubation. The nurse notes intense itching, a burning sensation and redness around the nose and ears, and swelling around the nostrils. The physician asks the pharmacist to investigate this adverse event. | The pharmacist identifies the presence of latex in the nasogastric tube, which might explain the symptoms. | The pharmacist recommends using a latex-free tube while waiting for an allergist to evaluate the situation. |
| **42** | Mr. L, 56, is an active smoker and has been hospitalized after exacerbation of COPD. He is receiving oxygen via a nasal cannula. To limit irritation, the physician prescribes a topical, oily substance. | Contact between an oxygen nasal cannula and an oily substance is contraindicated, due to the risk of burns. | The pharmacist warns the patient about the risk and recommends a suitable oil-free medical device. |
| **43** | Mr. J has been hospitalized for facial trauma. The surgeon orders a custom maxillofacial prosthesis directly from the manufacturer without informing the pharmacy. | During a phone call with the manufacturer, the pharmacist learns of the situation and identifies the need for sterilization of the implantable device. | The pharmacist informs the surgeon and proceeds with sterilization of the device. |
| **44** | Mr. J has been admitted to a rehabilitation ward and is using a sterile urine collection bag. However, there is no infection or cytological indication. | A non-sterile bag would provide the same benefit at a lower cost. | The pharmacist suggests switching to a non-sterile urine collection bag. |
| **45** | Mr. I is receiving long-term antibiotic therapy for multidrug-resistant Staphylococcus aureus endocarditis. The physician prescribes a double-lumen peripherally inserted central catheter. | The treatment regimen does not justify the use of a double-lumen device. | The pharmacist requests a new prescription specifying a single-lumen peripherally inserted central catheter. |
| **46** | Mr. H, 76, has moderate kidney failure and has had a colectomy. Patient-controlled analgesia with morphine is set up, with a basal rate of 2 mg/h and a bolus of 1 mg. | During the night, the nurse administered two boluses. The administration of boluses had not been discontinued because it was thought this would prevent pain on waking. The patient developed respiratory depression with bradypnea and desaturation. The administration of naloxone reversed the effects of the overdose. The pharmacist identified an error in the prescription: an order for nurse-administered IV bolus remained active after the initiation of patient-controlled analgesia . | The pharmacist requests the discontinuation of prescriptions of bolus morphine. |
| **47** | Mr. H, 76, has been admitted to a geriatric ward for a recurrent venous ulcer. During their rounds, the attending physicians observe that the wound has not improved, despite the use of dressings. | The pharmacist notes that although dressings were prescribed, compression therapy was not. | The pharmacist suggests adding a compression bandage. |
| **48** | Mr. H has undergone cataract surgery, with the placement of an intraocular lens. | During a predischarge medication reconciliation, the clinical pharmacist realizes the patient has not received a traceability document for the implant. | The pharmacist asks the operating room staff to retrieve the traceability information and generate an implant card. |
| **49** | Mr. G is being treated for multiple spinal fractures after a paragliding accident. The pharmacy receives a traceability form after surgery. | The operating room did not record the VERTAPLEX device used during the vertebroplasty on the traceability form. | The pharmacist asks the surgical ward to update the traceability data in the patient's electronic record. |
| **50** | Mr. C. has undergone partial prostatectomy and requires a urinary catheter after the operation. The physician prescribes replacement every two days, with no limit on the duration of treatment. | After several days, the pharmacist notes that the catheter is replaced every two days. The hospital's standard protocol indicates replacement every 7–14 days, except in the context of an infection or a specific indication. | The pharmacist recommends reducing the replacement frequency to every 7 to 14 days. |
| **51** | Mr. T is scheduled for a processor replacement for his cochlear implant. According to the present reimbursement rules, the processor cannot be replaced more frequently than every 5 years. | The prescribed reference is incompatible with the implant, which was placed 11 years previously. | The pharmacist suggests that a compatible processor should be prescribed. |
| **52** | In 2015, Mr. L had a vagal nerve stimulator (type 102R, dual connection) implanted. The battery is running down, and so the device needs to be replaced. The attending physician prescribes a single-connection stimulator. | Analysis and discussion reveal a mismatch between the prescribed medical device and the patient's needs. | The pharmacist and the physician validate the correct stimulator, and a new prescription is issued. |
| **53** | The case of a 3-month-old infant with severe bronchiolitis on non-invasive ventilation (NIV) is discussed during a staff meeting. Despite the presence of agitation and respiratory distress, the level of saturation is 100%. | The pharmacist suspects an excessively high FiO_2_ setting for the NIV. | The pharmacist recommends a gradual reduction in the FiO_2_. |
| **54** | During her visit to the obstetrics ward, a pharmacy technician notices that a non-NRFit® medical device has been used to treat Ms. T, who has recently been admitted to the delivery room. The department has recently switched to the exclusive use of NRFit® devices. | The use of a non-NRFit® medical device in a ward that has switched to NRFit®, as recommended by the hospital. | The pharmacist asks the nurse to return the products to the ward and to order NRFit® devices. |
| **55** | A pharmacy student reviews prescriptions for Mr. G, a patient who is immobile and remains seated all day. | During the reconciliation, the student notes the absence of an anti-bedsore cushion and reports this to the pharmacist. | The pharmacist suggests the prescription of an anti-pressure-ulcer cushion. |
| **56** | Victor, a baby born at 29 weeks of amenorrhea, has been admitted to the neonatal intensive care unit and is receiving a nonstandard, continuous infusion of insulin diluted to <1 IU/mL. | The use of a long PVC tube, a low flow rate and a low insulin concentration result in the loss (through adsorption onto the PVC) of up to 80% of the insulin. | The pharmacist recommends prescribing the hospital's standard protocol, in which the tubing is primed with insulin before administration; this limits adsorption and ensures appropriate delivery of the drug. |
| **57** | The pharmacy receives a traceability form after Ms. T has undergone unilateral breast reconstruction. | The prescription indicates that two breast implants were implanted during a unilateral reconstruction; in fact, one implant had been used as a sizer (i.e. a trial implant used to determine the appropriate size). | The pharmacist asks the ward to change the traceability status of one of the prostheses to "not implanted." |
| **58** | Noémie (aged six months) is scheduled to undergo placement of a sirolimus-coated stent. There are no literature data on the kinetics of sirolimus release in children. | The greater risk of toxicity requires closer patient monitoring. | The pharmacist recommends monitoring kidney and liver functions and, if necessary, therapeutic monitoring of the sirolimus. |
| **59** | Following a multidisciplinary staff meeting, a cobalt chrome prosthesis is prescribed for Mr. R. | The patient's medical records mention the result of a metal hypersensitivity test, indicating a cobalt allergy. | In view of this contraindication, the pharmacist contacts the physician and suggests the use of a different type of prosthesis. |
| **60** | Jeanne (aged 1 month and born after 33 weeks + 1 day of amenorrhea) is hospitalized with suspected meningitis. The patient's course on the ward is noted as follows:   - D+10: Bilateral subependymal and intraventricular hemorrhages undergoing resorption, with areas of leukomalacia. - Day 25: the hemorrhages have almost completely resorbed but multiple cystic cavities (associated with leukomalacia lesions) have appeared in the frontoparietal white matter.   🡪 A 10 mL/kg therapeutic lumbar puncture is indicated and will be continued if well tolerated/effective. | During a staff meeting, the pharmacist learns that the medical team plan to use a 20G spinal needle for the lumbar puncture.  The French national guidelines on the prevention and management of lumbar puncture injuries (HAS/SFAR, 2019) recommend the first-line use of pencil-point needles with a 22-27G tip. | The pharmacist suggests using a 25G pencil-point spinal needle with an introducer. |
