# Supplementary material for: Validation of an updated French national tool for reporting pharmacist interventions for all healthcare products
Source: Int J Clin Pharm. 2026 Jul 4;48(5):2015–25. doi: 10.1007/s11096-026-02181-5 (PMC13569509; doi:10.1007/s11096-026-02181-5)
Supplement: Supplementary file 2 [file 11096_2026_2181_MOESM2_ESM.docx]

**Online Resource 2: an updated version of the tool used in French hospitals to classify product-related problems (PRPs) and pharmaceutical interventions (PIs): English and French versions.** *This document shows the revised version of the tool. The tool comprises two grids: one for PRPs and the other for PIs. Each PRP or PI item is described in the right-hand column.*

| **Problem grid** | | |
| --- | --- | --- |
| **Item No.** | **Label** | **Details** |
| 1.1 | Contraindications or non-compliance with guidelines | - Non-compliance of the selected healthcare product with the hospital's formulary: an equivalent alternative listed in the formulary is available.  - Non-compliance in the selection or omission of a healthcare product with regard to validated consensus statements: according to consensus statements, recommendations, guidelines, and/or summary of product characteristics/device instructions for use, another healthcare product is equally effective and less expensive or less toxic for the patient in question.  - Contraindication to use of the healthcare product: for example, the patient has a condition that contraindicates the prescribed treatment (e.g. asthma and beta-blockers; MRI and incompatible implantable medical devices). |
| 1.2 | Nontreated indication | - Absence of treatment for a valid medical indication.  - A healthcare product was not prescribed following a care transition.  - The patient did not receive a prescription for prophylaxis or premedication.  - A synergistic or corrective medication should be added. |
| 1.3 | Subtherapeutic dosing/insufficiently frequent replacement | - Subtherapeutic dosing: the healthcare product is being used at too low a dose (per time period) for the patient in question.  - The treatment duration is too short (e.g., an antibiotic prescribed for 5 days instead of 10 days).  - Frequency of use or replacement is too low. |
| 1.4 | Supratherapeutic dosing/excessively frequent replacement | - Supratherapeutic dosing:  . The healthcare product is used at too high a dose for the patient in question.  . Drug accumulation is observed.  - Duplicate therapy: the same active substance is prescribed more than once (e.g., several pharmaceutical specialties containing paracetamol).  - The frequency of use or replacement is too high |
| 1.5 | Healthcare product not indicated | - A healthcare product is prescribed without a valid indication.  - A healthcare product is prescribed or used for an excessively long duration (e.g., two weeks of antibiotic therapy for a common condition; long-term negative pressure wound therapy prescribed without a valid indication).  - Therapeutic duplication: two drugs with different active substances but from the same therapeutic class are prescribed, resulting in pharmacological redundancy (e.g., tamsulosine and alfuzosine), or two medical devices are used together with redundant effects (multiple valves on the same infusion line) |
| 1.6 | Drug interaction | An interaction between two healthcare products decreases or increased their effects.  - According to the ANSM GTIAM classification, interactions are categorized as:  Interaction to be taken into consideration (depending on the clinical relevance), Precautions for use, Combination not recommended, Combination contraindicated.  - An interaction is reported in the literature but is not included in the ANSM GTIAM classification (bibliographic references should be provided). |
| 1.7 | Adverse drug reaction | The patient experiences an adverse event , even though the healthcare product is being used in compliance with the guidelines. The event may be evidenced by clinical, biochemical or pharmacokinetic variables. |
| 1.8 | Inappropriate administration route and/or use | The selected healthcare product is appropriate but the administration route is not.  - An alternative route would be more effective, more environmentally sustainable, or less costly with equivalent effectiveness  - The method of use is inappropriate (reconstitution, dilution, handling, or duration of use).  - Inappropriate choice of the pharmaceutical formulation (a liquid formulation for a patient who has difficulty swallowing, the avoidance of effervescent tablets with corticosteroids, or a formulation that is not compatible with enteral nutrition at home).  - Incomplete prescription (missing data on the duration, dosage, dimensions, etc.).  - A suboptimal administration schedule (dose timing and distribution).  - Inappropriate characteristics of the healthcare product (inappropriate material or reference selected).  - Inappropriate combination of healthcare products (e.g., devices used for an IV line setup). |
| 1.9 | Failure to receive the treatment:  • Lack of availability  • Non-compliance  • Physicochemical incompatibility | - Product not available:  Discontinuation of manufacture, suspension of the marketing authorization, suspension of CE marking, lack of stock, or shortage of supply.  - Compliance issues.  - Physicochemical incompatibility between injectable drugs and/or with a medical device, aerosols, oral drops, etc., with a risk of precipitation during administration.  - Incompatibility between two medical devices (e.g., a sound processor that is incompatible with the cochlear implant) . |
| 1.10 | Required monitoring not performed | The patient is not receiving appropriate or sufficient monitoring for the treatment or its discontinuation: monitoring of clinical, laboratory or pharmacokinetic variables (e.g., blood glucose, ECG, blood pressure, therapeutic drug monitoring)  Inadequate monitoring or lack of a consultation with a specialist. |
| 1.11 | Non-compliance with the healthcare product supply chain | Device not traceable (e.g., nontraceable blood-derived medicinal product or implantable medical device).  Incorrect or incomplete traceability information (e.g., incorrect batch number, reference, expiry date, or patient identifier).  Failure to use or install the healthcare product as specified.  Healthcare product traceability information not transferred to the electronic medical record.  Incorrect procurement pathway used. |

| **Intervention grid** | | |
| --- | --- | --- |
| **Item No.** | **Description** | **Details** |
| 2.1 | Addition (new prescription) | Addition of a healthcare product to the patient's treatment regimen |
| 2.2 | Treatment discontinuation | Removal of a healthcare product from the patient's treatment regimen, with no replacement. |
| 2.3 | Substitution/switching | Implementation of an alternative healthcare product, rather than the usually prescribed healthcare product:  - This may correspond to substitution with a generic (a market-related decision) or a substitution with a product listed in the hospital's formulary.  - Switching corresponds to the dispensing of an alternative product as part of an approved protocol.  - The alternative is better suited to the patient.  - The alternative can be offered when supply is disrupted. |
| 2.4 | Selection of the administration route | - A switch from IV administration to *per os* administration:  . A switch to a different product with equivalent efficacy and a transition from IV to *per os* administration.  . Switch from injectable to *per os* administration for the same product, with maintenance of efficacy.  - Selection of an administration route that is better suited to the patient. |
| 2.5 | Therapeutic monitoring | - Monitoring of the international normalized ratio, potassium level, clinical status, pharmacokinetic variables, etc.  - Request for or discontinuation of therapeutic drug monitoring.  - Request for or discontinuation of laboratory testing.  - Request for a consultation with a specialist. |
| 2.6 | Optimization of administration/use | - Medication administration plan:  . Distribution of dose timing in relation to meals or drug interactions, without changing the dose.  . Recommendations for optimal administration (e.g., on an empty stomach, between meals, in upright position, etc.).  - Clarification of administration instructions or prescription details (dose, reconstitution, dilution, duration of infusion, etc.).  - Advice on the correct use or prescribing of a healthcare product.  - Advice on selecting a healthcare product suited to the patient's clinical condition. |
| 2.7 | Dose adjustment | - Dose adjustment of a drug with a narrow therapeutic index, based on the drug concentration measured in a biological matrix, kidney function, liver function, or other laboratory results.  - Dose adjustment according to the patient's characteristics (weight, age, marketing authorization, or clinical situation).  - Extension of a treatment duration considered to be too short.  - Optimization of the frequency of use or replacement of a healthcare product. |
| 2.8 | Optimization of the healthcare product supply chain | - Addition or modification of traceability information associated with a healthcare product.  - Provision of traceability information to patients or healthcare professionals.  - Modification of procurement procedures for a healthcare product.  - Reminders about healthcare product supply conditions. |

| **Fiche problématique** | | |
| --- | --- | --- |
| **N° item** | **Libellé** | **Détail** |
| 1.1 | Non-conformité aux  référentiels ou Contre-indication | - Non-conformité du choix du produit de santé au livret thérapeutique :  Il existe un équivalent au livret thérapeutique.  - Non-conformité du choix ou omission d'un produit de santé au regard des différents consensus validés :  Un autre produit de santé est tout aussi efficace et moins coûteux ou moins toxique pour ce patient conformément aux consensus ou recommandations ou référentiels (résumé caractéristique : produit/ dispositif, notice d'utilisation).  - Il existe une contre-indication à l’usage de ce produit de santé :  Par exemple, le patient présente un terrain qui contre-indique le traitement prescrit :  asthme et bêtabloquant, IRM et dispositif médical implantable incompatible... |
| 1.2 | Indication non traitée | -Absence de thérapeutique pour une indication médicale valide.  - Un produit de santé n’a pas été prescrit après un transfert.  - Le patient n’a pas bénéficié d’une prescription de prophylaxie ou de prémédication.  - Un médicament synergique ou correcteur devrait être associé. |
| 1.3 | Sous-dosage/renouvellement insuffisant | - Posologie infra-thérapeutique : le médicament est utilisé à une dose trop faible pour ce patient (dose par période de temps).  - La durée de traitement est anormalement raccourcie  (Ex : antibiotique prescrit sur 5 jours au lieu de 10 jours)  - Fréquence d'utilisation/ de renouvellement trop faible |
| 1.4 | Surdosage/renouvellement excessif | - Posologie supra-thérapeutique :  . Le médicament est utilisé à une dose trop élevée pour ce patient.  . Il existe une accumulation du médicament.  - Un même principe actif est prescrit plusieurs fois sur l’ordonnance (ex : plusieurs spécialités pharmaceutiques contenant du paracetamol).  - Fréquence d'utilisation/ de renouvellement trop élevée |
| 1.5 | Produit de santé non indiqué | - Un produit de santé est prescrit sans indication justifiée.  - Un produit de santé est prescrit/ utilisé sur une durée trop longue  (Ex : antibiothérapie sur 15 jours pour une pathologie courante, thérapie par pression négative prescrite au long court sans indication).  - Prescriptions de deux médicaments à principe actif différent mais appartenant à la  même classe thérapeutique créant une redondance pharmacologique (Ex : tamsulosine et alfuzosine) ou deux dispositifs médicaux utilisés ensemble ayant des effets redondants (ex : utilisation de plusieurs valves positionnées sur une même ligne de perfusion). |
| 1.6 | Interaction médicamenteuse | Un produit de santé interfère avec un autre produit de santé et peut induire une  performance exagérée ou insuffisante.  - D’après le GTIAM de l’ANSM :  Association à prendre en compte (selon la pertinence clinique), Précaution d’emploi,  Association déconseillée, Association contre-indiquée.  - Interaction publiée mais non validées par le GTIAM de l’ANSM.  (préciser les références bibliographiques) |
| 1.7 | Effet indésirable | Le patient présente un effet indésirable alors que l'utilisation du produit de santé est conforme aux référentiels. Il peut s’agir d’un effet clinique ou biologique, cinétique. |
| 1.8 | Voie et/ou utilisation  inappropriée | Le produit de santé choisi est correct mais la voie d’administration n’est pas adaptée.  - Autre voie plus efficace, ou avec un moindre un effet sur l'environnement ou moins coûteuse à efficacité équivalente  - La méthode d’utilisation n’est pas adéquate (reconstitution, dilution, manipulation,  durée).  - Mauvais choix de galénique pour le médicament.(forme solution si difficulté à déglutir ou éviter le comprimé effervescent sous corticoïde ou forme non compatible avec la Nutrition entérale à domicile)  - Prescription incomplète (absence de durée, dosage, dimensions...)  - Plan de prise non optimal (répartition horaire et moment).  - Caractéristiques du produit de santé non appropriée (Mauvais choix de matériaux ou de référence)  - Association de produit de santé non adaptée (ex: dispositifs utilisés pour le montage d'une ligne de perfusion) |
| 1.9 | Traitement non reçu :  • Indisponibilité  • Inobservance  • Incompatibilité physico-chimique | - Non disponibilité de la spécialité :  Arrêt de fabrication, suspension d’AMM, suspension du marquage CE, rupture de stock, pénurie  - Problème d’adhésion thérapeutique  - Incompatibilité physico-chimique entre plusieurs médicaments injectables et ou avec un dispositif médical, aérosol, gouttes buvables… : risque de précipitation entre des médicaments incompatibles en cours d’administration.  - Incompatibilité entre deux dispositifs médicaux (ex : processeur de son et implant cochléaire non compatible) |
| 1.10 | Monitorage à suivre | Le patient ne bénéficie pas d’un suivi approprié ou suffisant pour son traitement ou sa déprescription : suivi biologique ou cinétique ou clinique (glycémie, ECG, tension artérielle, mesure de concentration d’un médicament…)  Surveillance/entretien non adéquat |
| 1.11 | Non-conformité au circuit des produits de santé | Traçabilité non réalisée (ex : médicament dérivé du sang ou dispositif médical implantable non tracé)  Traçabilité erronée ou incomplète (ex. numéro de lot, référence erronée, date de péremption erronée, identité patient erronée)  Échec d'utilisation ou de pose du produit de santé non spécifiée  Traçabilité du produit de santé non transférée dans Mon Espace Santé/ DPI  Mauvais circuit déclenché pour l'approvisionnement |

| **Fiche intervention** | | |
| --- | --- | --- |
| **N° item** | **Description** | **Détail** |
| 2.1 | Ajout (prescription nouvelle) | Ajout d’un produit de santé au traitement d’un patient |
| 2.2 | Arrêt | Arrêt d’un produit de santé du traitement d’un patient sans substitution. |
| 2.3 | Substitution /échange | Mise en place d’une alternative thérapeutique au produit de santé habituellement prescrit :  - Il peut s’agir d’une substitution générique (application de décisions liées à un marché) ou thérapeutique (formulaire local).  - L’échange thérapeutique correspond à la dispensation d’une alternative dans le cadre d’un protocole approuvé.  - L’alternative est mieux adaptée au patient.  - L'alternative peut être proposée dans le cadre de rupture/ tension d'approvisionnement |
| 2.4 | Choix de la voie  d’administration du médicament | - Relais voie injectable /voie orale :  . Alternative thérapeutique d’un produit différent à efficacité équivalente et passage voie injectable vers voie orale.  . Alternative voie injectable vers voie orale du même produit avec efficacité conservée.  - Choix d’une voie d’administration plus adaptée au patient. |
| 2.5 | Suivi thérapeutique | - Suivi INR, kaliémie, suivi clinique, suivi cinétique…  - Demande / arrêt du dosage d’un médicament.  - Demande / arrêt prélèvement biologique.  - Demande d'un avis auprès d'un spécialiste. |
| 2.6 | Optimisation des modalités  d’administration/ d'utilisation | - Plan de prise :  . Répartition des prises par rapport au repas ou aux interactions médicamenteuses sans modification de posologie.  . Conseils de prise optimale (ex : Prise à jeun, à distance des repas, en position debout…).  - Précisions des modalités d’administration ou du libellé (ex : Modalité de reconstitution, de dilution, durée d’une perfusion…).  - Conseil sur la bonne utilisation/ prescription d'un produit de santé prescrit  - Conseil sur le choix d'un produit de santé adapté à la situation clinique du patient. |
| 2.7 | Adaptation posologique | - Adaptation de la posologie d’un médicament à marge thérapeutique étroite en tenant compte d’un résultat de concentration de ce médicament dans un milieu biologique, de la fonction rénale et/ou de la fonction hépatique ou du résultat d’un autre examen biologique.  - Adaptation de la posologie d’un médicament par ajustement des doses avec le poids, l’âge, l’AMM ou la situation clinique du patient.  - Allongement d’une durée de traitement jugée trop courte  - Optimisation de la fréquence d'utilisation ou de changement d'un produit de santé |
| 2.8 | Optimisation du circuit des produits de santé | - Ajout ou modification de la traçabilité associée à un produit de santé  - Information au patient ou au professionnel de santé  - Modification des modalités de commande d'un produit de santé  - Rappels des conditions d'approvisionnements en produit de santé |
