# Supplementary material for: Validation of an updated French national tool for reporting pharmacist interventions for all healthcare products
Source: Int J Clin Pharm. 2026 Jul 4;48(5):2015–25. doi: 10.1007/s11096-026-02181-5 (PMC13569509; doi:10.1007/s11096-026-02181-5)
Supplement: Supplementary file 3 [file 11096_2026_2181_MOESM3_ESM.docx]

**Online resource 3. A case vignette illustrating the use of the revised Act-IP©.**

**Intravenous administration of an immunosuppressant and selection of an appropriate medical device**

A 58-year-old male patient was admitted to the intensive care unit for the management of multiple organ failure resulting from a severe bone infection. Combination therapy with rifampicin and levofloxacin was initiated for a planned duration of six weeks.

The patient's medical history included kidney transplantation five years previously and thus long-term treatment with an immunosuppressant (tacrolimus). Due to a deterioration in the patient's neurological status and the onset of swallowing difficulties, *per os* administration was no longer feasible; the tacrolimus had to be administered intravenously.

During preparation of the IV administration set-up, polyvinyl chloride (PVC) infusion tubing was initially selected.

**The pharmacist intervention**

**The product-related problem**

PVC infusion tubing may interfere with the adequate administration (bioavailability and effectiveness) of tacrolimus, due to the adsorption of the drug.

**Pharmaceutical intervention**

A pharmaceutical intervention was issued for replacement of the PVC tubing with a polyethylene extension set. Polyethylene is considered to be a more inert material that does not adsorb tacrolimus, thereby ensuring accurate drug delivery and optimal therapeutic exposure.

**Classification of the intervention, according to the updated Act-IP©**

Healthcare products involved: PVC infusion tubing and tacrolimus

| **Product related problem** | **Pharmaceutical intervention** |
| --- | --- |
| Failure to receive the treatment:  - Lack of availability  - Non-compliance  - Physicochemical incompatibility | Substitution/switching |
